# Biomedical research, a tool to address the health issues that affect African populations

**DOI:** 10.1186/1744-8603-9-50

**Published:** 2013-10-21

**Authors:** Emmanuel Peprah, Ambroise Wonkam

**Affiliations:** 1 Current address: National Institutes of Health, Building 1, RM 256A, Bethesda MD 20892, USA; 2 Division of Human Genetics, Faculty of Health Sciences, University of Cape Town, Anzio Road-7925, Observatory, Cape Town, South Africa

**Keywords:** Genomics, Biomedical, Research, Africa, Development, Policy

## Abstract

Traditionally, biomedical research endeavors in low to middle resources countries have focused on communicable diseases. However, data collected over the past 20 years by the World Health Organization (WHO) show a significant increase in the number of people suffering from non-communicable diseases (e.g. heart disease, diabetes, cancer and pulmonary diseases). Within the coming years, WHO predicts significant decreases in communicable diseases while non-communicable diseases are expected to double in low and middle income countries in sub-Saharan Africa. The predicted increase in the non-communicable diseases population could be economically burdensome for the basic healthcare infrastructure of countries that lack resources to address this emerging disease burden. Biomedical research could stimulate development of healthcare and biomedical infrastructure. If this development is sustainable, it provides an opportunity to alleviate the burden of both communicable and non-communicable diseases through diagnosis, prevention and treatment. In this paper, we discuss how research using biomedical technology, especially genomics, has produced data that enhances the understanding and treatment of both communicable and non-communicable diseases in sub-Saharan Africa. We further discuss how scientific development can provide opportunities to pursue research areas responsive to the African populations. We limit our discussion to biomedical research in the areas of genomics due to its substantial impact on the scientific community in recent years however, we also recognize that targeted investments in other scientific disciplines could also foster further development in African countries.

## Background

Advances in medicine using recent biomedical technologies, novel chemotherapeutic interventions and treatment regimens has allowed for rapid diagnosis and treatment of diseases, thus prolonging life for a significant number of people [1,2]. This is demonstrated by the substantial increase in the global life expectancy at birth in the past 20 years. Globally, male and female life expectancy has increased from 56.4 yrs and 61.2 yrs respectively to 67.5 yrs and 73.3 yrs in the decade from 1990 to 2010 [3]. In sub-Saharan Africa (SSA), substantial reductions in childhood mortality, through the introduction of life saving antiretroviral therapy and other preventative measures against communicable diseases are credited with the observed increases in life expectancy [3]. Whereas global life expectancy is the standard metric to measure the progress of countries toward combating disease resulting in prolonged life, another statistic, the healthy life expectancy (HALE) summarizes mortality and non-fatal outcomes of disease (e.g. longevity) in a single measure of averaged population health [4].

In 1990, countries with the lowest HALE for both sexes were in SSA except for Afghanistan [4]. Similar to life expectancy, HALE for a person at age 50 improved in the past 20 years (i.e. 1990 to 2010) for most countries, including many in SSA [4]. As increases in life expectancy occurred, years lived with disability (YLD) remained largely unchanged over the same 20-year period [5]. Life expectancy, HALE and YLD demonstrate that success has been mixed because: 1) as life expectancy has increased globally, countries in SSA are at the bottom of this and other measures and 2) the majority of diseases which have caused YLDs to remain relatively unchanged are non-communicable diseases (NCDs) and some communicable diseases (e.g. antiretroviral therapy for individuals have improved and increased the lifespan of many living with HIV/AIDS) [5].

With the observed increase in life expectancy, one could properly ask: Have genomic technologies contributed to this increase globally? If biomedical technologies, especially genomics have contributed to the increase in global life expectancy this would be one major justification for increased investment of these technologies in SSA. Although communicable diseases have decreased in SSA, the major challenges for the next few decades will include health issues associated with NCDs. Significant increases in YLDs for people living in SSA with well managed chronic conditions due to NCDs and infectious diseases will also become a challenge [5]. Biomedical science can play a role in combating the rise in NCDs and chronic conditions. For instance, biomedical science has the potential to improve the quality of life for many by identifying the optimal treatment regimens for several conditions [6-8]. Furthermore, the application of biomedical research to solve vexing problems has led to the creation of new fields of scientific inquiry and expansion of other areas spurring innovation [9,10]. In fact, publication of the human genome sequence has revolutionized medicine allowing the characterization of novel disease-related genetic variants. In this capacity, genomics (i.e. elucidating disease states by using technologies that characterize human and also non-human genomes) can be a very useful tool to further address communicable and non-communicable diseases in SSA because investments in biotechnology, especially genomics, have significantly impacted other regions. Although investments in genomic technologies have increased in other regions of the world, scientists in low- to middle-income countries that could utilize these technologies to improve health and to promote equity are concerned that the commercial development of genomic technologies will widen the gap between the developed and the developing world [11]. This concern was recognized by the World Health Organization (WHO) over a decade ago; the WHO commissioned the report *Genomics and World Health* in recognition of the potential and significant positive and negative impacts genomics could have on global health [12]. A decade after the WHO report was published, large-scale investments in biotechnology and human genomic studies have been made by Mexico, India, China and Thailand producing both economic and scientific dividends which we suggest have had a beneficial effect on life expectancy [2,13-15]. Within these countries an increase in life expectancy has been observed which can be attributed to medical innovation sparked by biotechnology and genomics (large scale vaccination programs), non-medical programs (continued education, improved literacy rate of the population) and other factors [16]. The factors necessary for the development and sustained utilization of large scale biotechnology including genomics, to develop sustainable economies are thoroughly discussed by Singer, Daar and colleagues [2,13,17,18]. This paper will express similar themes and focus on how biomedical research can address communicable and non-communicable diseases in Africa.

## Communicable diseases

Globally, deaths from communicable diseases, and maternal, neonatal and nutritional causes decreased by ~ 2.7 million from 1990 to 2010 (from 15.9 million deaths to 13.2 million deaths respectively) [19]. Notwithstanding, this observed decrease in deaths from communicable diseases, Southeast Asia and Africa together bore 54% of the total global burden of disease while accounting for only 40% of the world’s population [20]. With the significant decrease in deaths from communicable diseases, SSA still accounts for a significant burden of communicable disease worldwide [20]. Interestingly, the decrease in deaths from communicable, maternal, neonatal, and nutritional causes was due to significant reductions in several diseases (i.e. diarrheal diseases, lower respiratory infections, neonatal disorders, measles and tetanus) which reflect scaling up of effective treatments and utilization of technologies to combat these disorders associated with poverty [19]. Other communicable diseases including HIV/AIDS and malaria, contributed to a massive increase in deaths from 1990 to 2010; however, this increase did not affect the overall decrease observed from aggressive scaling up of medical interventions for children (0–12 yrs), which were the major age group that died from communicable diseases [19]. The significant reduction in child deaths associated with communicable diseases has increased universal life expectancy.

Biomedical and genomic technologies have been used to derive host-parasite relationships (e.g. between human-parasite, parasite-vector, and human-vector interactions) that have significantly increased our understanding of these complex interactions [21,22]. Traditionally, fieldwork has occurred in SSA to sample zoonotic vectors, parasites and infected humans in the areas where outbreaks have occurred. Fieldwork has allowed sample collection resulting in the genomes of *Plasmodium falciparum*[23], *Trypansoma brucei*[24], *Leishmania major*[25], and *Schistosoma mansoni*[26] being sequenced with additional molecular, biochemical and bioinformatics analyses [27]. For instance, data acquired from those scientific endeavors have been used to develop new therapies for schistosomiasis [28], trypanosomiasis [29] and malaria [30], using both genomics and traditional approaches, which have decreased the burden of disease in SSA. Currently, researchers are testing combinations of malaria treatments to find regimens suitable for children and also to identify the development of drug resistance in malaria [6]. Another technology, the genome-wide associations study (GWAS), has been used to provide genetic candidates for the development of control measures against malaria [6,31]. Samples collected during outbreaks have yielded new discoveries about both the infectious agents and population susceptibilities. Moreover, systematic analysis of samples collected during outbreaks (e.g. large vector and parasite collections) has allowed for determination of parasite susceptibility using pharmacological compounds [32]. In the future, investigation of genetic diversity of both vector and parasites within the environment using genomics will shed light on the distribution, epidemiological importance and evolutionary history of malaria vectors in the African rainforest [33]. Many of these new efforts, in which genomic technologies were utilized to investigate malaria and other infectious diseases, are being led by African investigators in Africa and they include chemotherapeutic interventions [6,33-35]. Publications highlight the emerging evidence that African scientists, with support from local and international partners, are starting to set a research agenda for the continent. The aforementioned examples showcased in various publications include: 1) improved diagnosis, 2) scaling up of treatment, 3) elucidation of the development of drug resistance in unicellular organisms, 4) elucidation of human genetic variants that affect drug metabolism, and 5) development of genomic tools for population-level analysis of parasite and human [14,36]. In the field of infectious disease, application of genomics technologies has been instrumental in the elucidation of the complex biology of infectious agents and how they interact with hosts [37].

In addition to malaria, African scientists have used genomic technologies (such as GWAS) to characterize HIV/AIDS in SSA, which has gained significant attention from researchers inside and outside SSA [7,38]. Genomic analysis of both African and non-African populations have found that several genetic variants (e.g. chemokine receptors, HLA, mannose-binding receptor and other genes) can provide increased resistance or susceptibility to HIV infections (Table 1) [7]. Africa could offer more research opportunities on HIV explicating the roles of these variants in HIV infections to improve prevention, care, and treatment prospects for individuals at risk of the disease [39]. Table 1 provides examples of GWAS and other studies pertaining to HIV, malaria, and tuberculosis which used mostly African and African ancestry populations (e.g. African Americans in the United States). Population-based genomic analyses has produced data about genetic susceptibility to disease and illuminated population-specific preventive approaches that could decrease HIV infections. Similar to the emergence of African investigators in the field of parasitology, many investigators on the African continent continue to elucidate HIV biology, treatment, transmission and also the effects of HIV on culture, social stigma and other negative societal issues of the disease [40-42].

**Table 1:** GWAS of African and African ancestry populations on communicable diseases obtained from the National Human Genome Research Institute catalogue of published genome-wide association studies (GWAS)

**Disease**	**Sample size**	**Replication sample size**	**Genes**	**Ref**
HIV (mother-to-child transmission)	100 Malawian infant cases, 126 Malawian infant controls	NR	NS	[43]
HIV-1 susceptibility	848 Malawian cases, 531 Malawian controls	NR	*AL591509.5, GLRX3, TXNL3, FAM174B, AC009271.7, ZDHHC19, BUD13*	[44]
HIV-1 viral setpoint	496 HIV-1 infected individuals of African ancestry; 302 HIV-1 exposed and uninfected individuals of African ancestry	NR	*VEGFC, EPHA5, HIST1H4A, AL391500.13, KB-67B5.12, CMTM8, AC095058.3, AC087190.5-2, DNAJC5B, ZFP90, MAD2L1, HSP90AB3P, Intergenic chr 2, GALNT14*	[45]
Malaria	958 Gambian cases; 1,382 Gambian controls; all children	1,087 Gambian cases; 2,376 Gambian controls, all children	*HBB, SCO1, DDC*	[31]
Tuberculosis	921 Ghanaian cases; 1,740 Ghanaian controls, 1,316 Gambian cases; 1,382 Gambian controls	1,226 Ghanaian cases; 3,825 Ghanaian controls; 236 Malawian cases; 779 Malawian controls; 332 Ghana parent/child trios and duos	*GATA6,CTAGE1,RBBP8,CABLES1*	[46]

NR not replicated, NS not significant.

We highlight genomics- and biotechnology-related research that was used to characterize and comprehend communicable diseases and demonstrate that this research has also led the way for substantial advances and improvements in the health of populations in SSA. Many countries (e.g. India, Brazil and China) have addressed communicable diseases by developing robust biotechnology industries [47,48]. Development of biotechnology (e.g. genomic research, and biopharmaceuticals) allows countries to benefit economically by lowering the cost of health-related products for local populations [48]. Globally, development of biotechnology has been a factor in increasing health and life expectancy in SSA, so we argue that a substantial investment in this area will produce significant benefit for SSA. We suggest that a similar model focused on chronic conditions could substantially mitigate the increases in NCDs as reported by the *Global Burden of Disease 2010* report [37,49,50].

## Non-communicable diseases

Communicable diseases account for the plurality of deaths in many African countries; however, there is a need to increase biomedical research in the areas of NCD. The burden of NCDs (e.g. heart disease, diabetes, some cancers and pulmonary disease) are expected to double in low- and middle-income countries within the next few decades [51]. The number of deaths from NCDs rose in 2010 by almost 34.5 million globally as deaths caused by communicable diseases decreased [19]. It is predicted that the healthcare systems of these countries will not be able to support the treatment of NCDs (e.g. Sickle Cell Disease (SCD)) especially in African countries in which the health infrastructure is taxed by infectious diseases [51]. The increase in chronic diseases would suggests that a realignment of priorities is needed in which substantially greater focus should be on mitigating the projected increases in NCDs in SSA. We argue that genomic technologies can benefit African populations in the area of non-communicable diseases by briefly discussing the knowledge scientists have gained about NCDs using these technologies.

GWAS and other genomic technologies have had considerable influence on the identification of genetic variants associated with NCDs. Several GWAS on NCDs have been conducted with African and African ancestry populations. Asthma is a complex NCD that significantly affects both the young and old [52]. In the countries in which asthma has be explored, many have reported that hospitalizations for asthma are 2–3 times higher for African and African ancestry populations compared to other ethnic groups [52,53]. GWAS exploring the genetic basis of disease in African ancestry populations have identified several genes associated with asthma susceptibility (Table 2). GWAS have found that different genetics variants were associated with asthma susceptibility in children of European and African ancestry suggesting different biological mechanisms could be at the basis of asthma susceptibility in these diverse populations [54,55].

**Table 2:** GWAS on non-communicable diseases in African and recent African ancestry populations obtained from the National Human Genome Research Institute catalogue of published genome-wide association studies (GWAS)

**Disease/Trait**	**Sample size**	**Replication sample size**	**Genes**	**Ref**
Asthma	422 cases; 1,533 controls	3,750 white cases; 13,365 white controls; 592 white trios; 1,903 black cases; 2,432 black controls; 929 black family members	*PDE4D*	[55]
Asthma	464 African American cases; 471 African American controls; 1,028 African Caribbean family members	994 European descent cases; 1,243 European descent controls; 2,331 African descent cases, 2,874 African descent controls (includes family members)	NS	[56]
Asthma	793 European ancestry child cases; 1,988 European ancestry child controls	917 European ancestry child cases; 1,546 European ancestry child controls; 1,667 African American child cases; 2,045 African American child controls	*DENND1B, CRB1*	[54]
Asthma	2,088 European American cases; 1,612 African American and African Caribbean cases; 1,688 Latino cases	2,727 European American cases; 2,147 African American and African Caribbean case; 2,299 Latino cases	*GSDMB, IL1RL1, TSLP, IL33, PYHIN1, C11orf71, CRCT1*	[57]
Sickle cell anemia (severity)	177 African American severe patients; 1,088 African American mild patients	68 severe patients; 95 mild patients	NS	[58]
Sickle cell anemia (HbF modifiers)	2040 African American sickle cell anemia patients	NR	*BCL11A, HBS1L-MYB*	[59]
Podoconiosis	194 cases and 203 controls from Africa	202 family trios (two parents and one affected child) for family-based association testing; 94 cases and 94 controls for HLA-typing	*HLA-DQA1*	[60]

NR not replicated, NS not significant.

Unlike asthma, the primary genetic variant underlying SCD has been identified, but the tools of GWAS are being used to determine the nature of the genetic modifiers associated with phenotypic diversity in SCD. SCD is the most common monogenic disease, occurring at its highest frequency in SSA [61]. As an illustration, people suffering from SCD will increasing effect public healthcare systems in SSA; it is predicted that 312,000 neonates with SCD are born globally with ~240,000 of those births being in SSA [62]. The combined populations of births of heterozygous and homozygous carriers of the SCD gene mutation HbAS is estimated at ~3.6 million and for HbSS ~240,000, respectively, producing an estimated ~4 million live births per year with SCD gene mutation [62]. In this capacity, SCD and other hemoglobinopathies represent a health burden comparative to that of communicable diseases in SSA [63]. An example of effective application of genomic technology for SCD in SSA could be the wide scale early diagnosis and preventive treatment of SCD, along with other hemoglobinopathies and monogenic disorders. The burden of SCD is disproportionately located in Africa; thus, prioritizing diagnosis, treatment and prevention for the low- and middle-income countries would be an effective means of bridging the gap between these and high-income countries. Furthermore, introduction of preventive genetic methods such as the utilization of DNA technology in childhood disease screenings to identify causal genetic variants would be beneficial to populations in SSA and considered an appropriate point of entry for genomic technologies [11]. An example that occurred in SSA is a Cameroonian center that recently introduced detection for SCD; this service offers reproductive options to families [64].

Moreover, genes implicated in SCD severity have been shown to modulate fetal hemoglobin (HbF) levels and were confirmed by GWAS in African and African ancestry populations [65]. A study showed that the 3 loci described in other populations that affect HbF levels and clinical severity has a significant impact in Tanzanian patients [66]; however, some variants affecting HbF in Tanzanians are not well represented in European populations. This and other similar findings suggest that there is a need to search for additional loci through independent GWAS in African populations [66]. Identifying loci that affect HbF levels and, consequently, disease severity could be used to anticipate one’s ability to produce HbF from birth, and plan in advance the introduction of treatment (e.g. hydroxyurea, a drug which has been shown to increase concentrations of HbF) to reduce the negative impact of SCD, which could include pain crises, acute chest syndrome and severe anemia resulting in blood transfusions [67]. Interestingly, evidence supports asthma and SCD as distinct co-morbid conditions in which the underlying mechanisms of phenotypic variations of the two diseases might be similar [68-70]. Table 2 lists GWAS studies that have found associations for genetic variants in NCDs. For both asthma and SCD, the complexity of the phenotype would suggest that genetic heterogeneity is a major contributor to the clinical severity in both diseases. Furthermore, the diversity within African populations can lead to a comprehensive characterization of genetic variants associated these both asthma and SCD.

Another NCD, Podoconiosis also called non-filarila elephantiasis of the lower leg, occurs in SSA because of environmental exposure to soil. Recently, family-based association study using GWAS was conducted to elucidate the genetic underpinnings of podoconiosis. This study, focused on continental African population groups, found genetic susceptibility to podoconiosis [60]. In addition to identifying genes associated with NCDs such as podoconisosis, GWAS has been used to identify treatment outcomes based on genotypes for NCDs. Overall; genomics-related biomedical research has given a better picture of the genetic underpinnings and molecular biology of many of these diseases which affect SSA populations.

The *Global Burden of Disease 2010* reported substantial increases in cardiovascular disease; ischaemic heart disease, stroke and chronic obstructive pulmonary disease were among the leading causes of death globally [19]. In SSA, many have recognized that cardiovascular diseases are a significant contributor to mortality; for example, in Kenya, acute myocardial infarctions are not uncommon [71]. Tanzania has observed a significant increase in the number of deaths for cardiovascular disease (from 16% in 2003 to 24% in 2007) [72]. In hypertension, genetic variants found in African ancestry populations are associated with differential responses to antihypertensive drugs (*e.g.* thiazide diuretics) commonly used to regulate blood pressure [73]. These genetic variants offer ways to distinguish good responders (patients that show improvement on diuretics) from non- or poor-responders (patients with a negative response to diuretic mediations for the treatment of hypertension) which provide information on how best to treat hypertensive patients. Because of this escalating dilemma faced by low- to middle-income countries in SSA, considerable resources will be needed to address the observed and impending ballooning of NCDs (e.g. hypertension and cardiovascular diseases) in the coming decades [19,72].

The observed increases of NCDs for the past 20 years (1990 to 2010) are due to factors that include demographic changes leading to a rise in the proportion of people older than 60 years [4,19]. This continued increase in life expectancy has not been homogeneous globally among age groups [3]. The heterogeneous distribution of life expectancy globally has resulted in an increase in people living longer with chronic conditions in SSA [74]. The scale of the challenge posed by the combined and growing burden of chronic infectious diseases and NCDs, including genetic conditions, demands an extraordinary response that few SSA countries currently provide. With the increase in life expectancy due to positive treatment outcomes and increased prevention of disease, SSA will increase its populations of the chronically ill in the coming decades. This situation is illustrated by WHO data indicating that life expectancy has risen; however, YLDs has remained constant globally [5]. We suggest that biotechnology and genomics can be used to address many of the issues associated with NCDs, similar to their use in infectious disease research. Also, a gradual increase in resources available to understand NCDs, while also addressing communicable diseases, is needed [75]. Essentially, we envision a model in which, as the burden of infectious diseases decreases, would create an opportunity to increase research focused on NCDs.

## Challenges for genomics research in Africa

The present state of genomic research in Africa is in its infancy; nevertheless, substantial biomedical research continues to occur in the areas of agriculture, genetics, biomedical science and several other areas [17]. Excluding South Africa, research funding amounts to < .5% of GPD indicating that the majority of funding for scientific research, training and infrastructural development occur through support from high-income countries [76]. Many have suggested that the influx of donor money to support African research has had a negative impact in which relatively wealthy nations (e.g. Nigeria and Angola) do not invest in funding to support biomedical research [77-79]. However, it must be acknowledged that significant infrastructure exists in Africa for biomedical research. The challenges that exist for genomics research in Africa include improving infrastructure, and creating and maintaining sustainable collaborations that are currently present on the continent.

Infrastructure for various research centers has been created with financial and technical investment from the United Nations, world governments, and biotechnology companies with business interests on the continent [13,80]. Currently infrastructure exists in South Africa, Egypt, Tunisia, Nigeria and Kenya [81]. These centers have also made substantial investments in agricultural science compared to other Africa countries and are increasing their research capacity [81]. Recently, African countries have started to realize the necessity of developing a vibrant research infrastructure from local government investments [51]. To address the issue of local investment, members of the African Union (AU) of countries have agreed to steadily increase funding for research until it reaches at least 1% of GDP within the next few years [76]. This is a significant development and it demonstrates that AU partner nations understand the value of research as a potential mechanism to: 1) decrease the burden of disease that affects their populations, 2) create cheaper and “home grown” alternatives to pharmacological agents that are usually purchased from high-income countries, 3) acknowledge the economic benefits that investments in research infrastructure can develop through home-grown companies, which in turn provide jobs to citizens who eventually pay taxes [2]. Local investments have occurred in many countries in the Southern Hemisphere allowing these countries to improve their economies which could be suggested as a way forward for SSA [2,15,18].

A second challenge for genomics research in Africa concerns research priorities of African countries and the high-income countries which fund and support research. Research priorities between high-income countries (i.e. United Kingdom, USA) and low- to middle-income African countries differ in many areas [81]. In addition to the differences, because SSA countries have a significant communicable disease burden, and disproportionately have more individuals affected by YLDs, African led research endeavors could significantly reduce the mortality associated with those diseases [5,76,79]. The differences in research priorities between African countries and high-income countries is illustrated by the lack of attention to diarrhoeal diseases (DD) and lower respiratory infections (LRI), which cause significant mortality in children in SSA [5]. In 2006, only 26 clinical trials occurred for DD and LRI (20 for DD and 6 for LRI) compared to 205 for malaria [81]. This suggests the need for further development of resources for African scientists to develop their own research priorities. Case studies in Cameroon and Tanzania illustrate the potential of genomic research in SCD and its applications in public health interventions. More studies to characterize genomic variations that affect phenotypes of SCD and their application in primary and secondary preventions, as well as prospective studies on genomic-based therapeutic interventions, could allow Africa, where two-thirds of SCD patients live, play a major global role in hemoglobinopathies research. With a significant decrease in communicable diseases observed in the past 20 years for SSA, a monumental shift has begun, suggesting a realignment of goals and priorities for the next few decades [4,5,19]. This realignment of priorities will occur as SSA considers how best to address the ageing population.

The third challenge is overcoming the scientific diasporas that have emerged between low- to middle-income countries compared to high-income countries [50,82]. The large migration of physicians, nurses, scientists and other highly educated professionals to high-income countries termed, “brain drain”, is of considerable significance to economic development [82]. African countries are placed in a disadvantaged position for technology development because professional, highly educated cadres of people are needed to develop, operate and maintain these technologies and lead scientific endeavors. This migration was illustrated in a 2004 report which stated that the import of highly qualified medical professionals from Ghana saved ~ £103 million (~$200 million) in training costs for the National Health Service in the United Kingdom [83]. From this observation, some have argued compensation for the country of origin was needed to offset the loss of this important and critical human capital [84]. Currently, in many regions of Africa with stable economies, migration is due to low salary for professionals [49]. The migration problem is prevalent in medicine and other scientific fields [85]. Several groups have suggested that brain drain is one major cause of scientific diasporas between industrialized and emerging nations [50]. Other groups suggest that the lack of infrastructure, economic development and governance are issues that must be addressed and brain drain is a symptom of these problems [18]. To address how these problems have arisen, it is important to examine the scientific diasporas in a new paradigm in which engagement of the continent, with equal partnership in developing collaborative research priorities which are also associated with economic development is paramount [86].

This scientific diasporas can be overcome via several mechanisms [50]. Even-though these mechanisms do not replace the loss in human capital, the mechanisms can mitigate losses through: 1) support of local foreign scientists, 2) establishment of equal partnerships in collaborative efforts between foreign-born scientists residing in industrialized countries with leaders and scientists of their countries of origins, 3) specific targeted compensation to build genomics/biomedical/health infrastructure, and 4) technology transfer among public and private sectors, which have been shown to be successful in many countries [86]. Within this framework, a balance will be needed for providing incentives for individuals to return to their country of origin versus training scientists and developing infrastructure. We must also acknowledge that corruptions and/or mismanagement of these well-intentioned programs can perturb any advances; however, a multifaceted approach would be important to overcome the barriers expressed in the first challenge. We advocate a multitiered approach because it has been shown to have success in South and Central America and in Asia [13,15]. The multitiered approach allows for investment in several areas while also balancing competing interests. The situation in SSA is quite challenging; nonetheless, if the recommendations are implemented within SSA success is also possible [13,18,87].

Overall a positive perception of research conducted by African scientists in SSA is gaining momentum, but it will take several years for this new paradigm to be the norm by which research occurs in Africa. The aforementioned challenges are not exhaustive but are significant barriers to development of biomedical technologies. A significant need to establish and implement ethical guidelines that allow international researchers conducting research in Africa to create capacity and also to help train the next generation of African scientists should be addressed. These issues are not only for the international community but also for African scientists who have significant obligations to develop the research infrastructure and culture within their own countries. For example, several areas that could be addressed without significant international coordination include collaboration amongst African scientists in Africa. African scientists could increase collaborations in areas of training, education and research which in turn will increase synergies in biomedical and genomic research producing a lower cost for everyone involved. These among other factors present unique opportunities for developing biomedical research emphasizing genomics within Africa.

## Conclusion

The challenges and potential to address the health issues of African populations are not unique to SSA because many countries (e.g. India, Mexico, Brazil and China) have used the development of biomedical research technologies including genomics to begin addressing the health issues of their respective populations [2,13]. In SSA, the Republic of South Africa has embraced many of the tenants of globalization including supporting the developing biotechnology sector [87]. In theory using biomedical research to develop scientific capacity in Africa could significantly reduce the migration of intellect from low- to high-income countries. Development of biomedical research including genomics research in Africa could provide networking opportunities for African research scientists, clinicians, social scientists and bioethicists. Similarly, networking by African scientists from different fields was instrumental in the foundation of several research consortia (e.g. MalariaGEN, and HapMap). This collaborative spirit has also sponsored African students (potential research leaders) and African scientists from the Diaspora to attend annual meetings in Africa [88,89]. Such African Diaspora participation generates collaborations with local African scientists working in Africa that could ultimately mitigate brain drain by fostering interaction [90]. The issue of brain drain cannot be solved without budgetary commitment from SSA countries which stand to significantly benefit from the reduction in migration from SSA to high income countries.

With the budgetary constraints of many low- to middle-income African economies, committing funds to support biomedical- and genomics-related research might be viewed by some as an esoteric exercise. The immediate needs for medications to prevent infectious and non-infectious diseases in many regions of Africa are paramount but should not obscure the future health needs of the population. We argue that in addition to addressing the health needs of the population in the present, a vision for the future is also needed. This vision should include addressing the growing burden of NCDs while also recognizing the need to develop policies which promote economic development through science and biotechnology development. The needs of the populations in SSA can be addressed while contemporaneously building a future based on intellectual investment in people using models from other countries. The research needs for future populations start in the present in which biomedical research, including genomics could be indispensable. In terms of economics, biomedical research (such as genomics) offers ways to foster home grown development, manufacturing, and diagnosis and also inform on the delivery of healthcare. There is a tremendous opportunity for biomedical research to flourish in Africa based on examples by many South/South collaborations; furthermore, strong government commitment must be made for the promise of biomedical research to be delivered to the African continent.

## Competing interests

The authors declare no competing interests.
